# A Quinoline-Benzimidazole Probe for Efficient Detection of Imidacloprid: Mechanisms and Applications

**DOI:** 10.3390/molecules31172992

**Published:** 2026-08-26

**Authors:** Hua-Fen Wang, Jing Zhu, Ye-Wu He, Man Wang, Jia-Xiang Zhang, Yu-Wei Zhuang, Zhi-Guang Suo, Sheng-Qiang Zhou, Yan-Chang Zhang, Hai-Jiao Xie

**Affiliations:** 1 High & New Technology Research Center of Henan Academy of Sciences, No. 56 Hongzhuan Road, Zhengzhou 450002, China; 2 Henan Key Laboratory of Cereal and Oil Food Safety Inspection and Control, College of Food Science and Technology, Henan University of Technology, Zhengzhou 450001, China; 3 The Material Research Institute of Henan Academy of Sciences, Zhengzhou 450046, China; 4 Hangzhou Yanqu Information Technology Co., Ltd., Y2, 2nd Floor, Building 2, Xixi Legu Creative Pioneering Park, No. 712 Wen’er West Road, Hangzhou 310003, China

**Keywords:** fluorescent probe, imidacloprid detection, quinoline-benzimidazole, proof-of-concept

## Abstract

To explore an alternative detection approach for the pesticide imidacloprid (IMI), this study repurposed the quinoline-benzimidazole fluorescent probe **DQBM-B**—previously developed for Co^2+^ recognition—and investigated its detection performance and interaction mechanism toward IMI in the aggregated state. The optimal working conditions of the probe were determined by optimizing key detection parameters, and the sensing performance and matrix compatibility were evaluated through selectivity tests and proof-of-concept spiked cucumber extract analysis. The **DQBM-B** aggregates interact with IMI synergistically through intermolecular hydrogen bonding and π–π stacking, which enrich IMI at the aggregate surface to create a local enrichment layer. The observed fluorescence quenching arises from the synergistic contribution of static quenching (due to ground-state complex formation) and the inner filter effect (IFE). Under the optimal conditions, the system exhibited a detection limit of 0.75 μmol L^−1^ for IMI with favorable anti-interference ability. The matrix effect evaluation in cucumber extract demonstrated good recovery and precision, demonstrating the feasibility of the aggregation-regulated IFE strategy in complex food matrices. This study expands the application scope of the **DQBM-B** probe from metal-ion sensing to pesticide detection and provides a metal-free, aggregation-regulated strategy for the fluorescence detection of neonicotinoid pesticides.

## 1. Introduction

As one of the most widely used neonicotinoid insecticides worldwide [1], imidacloprid (IMI) has been extensively applied in pest control in agriculture, forestry and animal husbandry due to its high systemicity and insecticidal activity [2]. IMI exerts its toxicity by selectively acting on insect nicotinic acetylcholine receptors and blocking neural signal transmission. However, IMI is environmentally persistent to a certain extent, and its residues in agricultural products pose potential threats to the balance of ecosystems, and also adversely affect human health through the food chain [3,4,5]. Therefore, the rapid, sensitive and accurate detection of IMI has become a research focus in the fields of environmental monitoring and food safety control.

At present, the common detection methods for IMI mainly include chromatography-mass spectrometry (GC–MS, LC–MS/MS), enzyme-linked immunosorbent assay (ELISA), electrochemical sensors, and optical sensors. Chromatographic techniques, particularly LC–MS/MS, remain the gold standard for IMI quantification due to their high sensitivity, accuracy, and ability to simultaneously detect multiple residues [6]. However, chromatographic methods rely on expensive instrumentation, skilled operators, and tedious sample pretreatment, making them unsuitable for on-site rapid screening. As alternative approaches, electrochemical sensors offer portability and low cost, as demonstrated by Brahim et al. [7] for IMI detection on a boron-doped diamond electrode. Nevertheless, electrochemical sensors often exhibit limited selectivity and are susceptible to electrode fouling in complex matrices. Similarly, immunoassays such as the colloidal gold immunochromatographic method developed by Wang et al. [8] enable rapid screening, yet they suffer from antibody instability and false-positive issues.

Fluorescence sensing technology has emerged as a promising alternative for pesticide residue detection due to its simple sample pretreatment, fast response, and high sensitivity [9]. In recent years, various fluorescent probes for IMI detection have been developed based on different recognition strategies. Metal-ion-mediated fluorescent probes represent a major class of reported systems. For example, Radhakrishnan et al. [9] developed nitrogen-doped graphitic carbon dots for the fluorometric detection of copper and IMI. Ud Din Mir et al. [10] constructed a Zr(IV) metal–organic framework for fluorometric detection of IMI in environmental water and biological fluids. Llorent-Martínez et al. [11] reported a luminescent probe based on terbium-carbon quantum dots for IMI quantification in caneberries. While these metal-complex-based probes demonstrate promising sensitivity, they inherently depend on the presence and stability of metal ions, which may suffer from ion interference, metal leaching, and environmental toxicity concerns. Moreover, most of these systems operate via single-wavelength intensity changes, which are vulnerable to environmental fluctuations and instrumental variations [6].

As summarized in Table 1, most reported fluorescent sensors for IMI rely on metal-containing nanomaterials or metal-ion mediation.

In contrast, metal-free fluorescent probes based on pure organic fluorophores offer advantages of environmental friendliness, structural tunability, and freedom from metal-ion interference; however, the development for IMI detection remains relatively scarce. Recently, aggregation-induced emission (AIE) strategies have attracted considerable attention in sensor design [14,15], as the aggregate state can create local microenvironments that enrich target analytes and amplify signal responses through mechanisms such as the inner filter effect (IFE) [16]. For instance, π-conjugated aromatic systems can form ordered aggregates via π–π stacking [17], generating enrichment interfaces that enhance analyte-probe interactions. Despite these advances, the rational design of non-metal fluorescent probes for IMI detection, particularly those leveraging aggregation-state regulation and IFE-based signal transduction, remains largely unexplored.

It is noteworthy that fluorescent probe scaffolds inherently lend themselves to multi-target sensing. Probes based on scaffolds such as rhodamine, BODIPY, and coumarin have been widely demonstrated to detect diverse classes of analytes—from metal ions to neutral organic molecules—through tailored structural modifications and distinct recognition mechanisms [18,19,20,21]. For instance, rhodamine-based probes have been repurposed from metal-ion sensing (Fe^3+^, Cu^2+^, Hg^2+^) [20] to the detection of reactive oxygen and nitrogen species (H_2_O_2_, NO, ClO^−^) by altering the recognition moiety while retaining the same fluorophore core [21]. Similarly, BODIPY derivatives have been applied to both Cd^2+^ detection and organochlorine pesticide (dicofol) sensing [19]. Inspired by this versatility, the present work extends the quinoline–benzimidazole scaffold of **DQBM-B** from Co^2+^ coordination-based sensing to IMI detection via aggregation-regulated non-covalent interactions and Inner Filter Effect (IFE) amplification.

**Figure 1:**
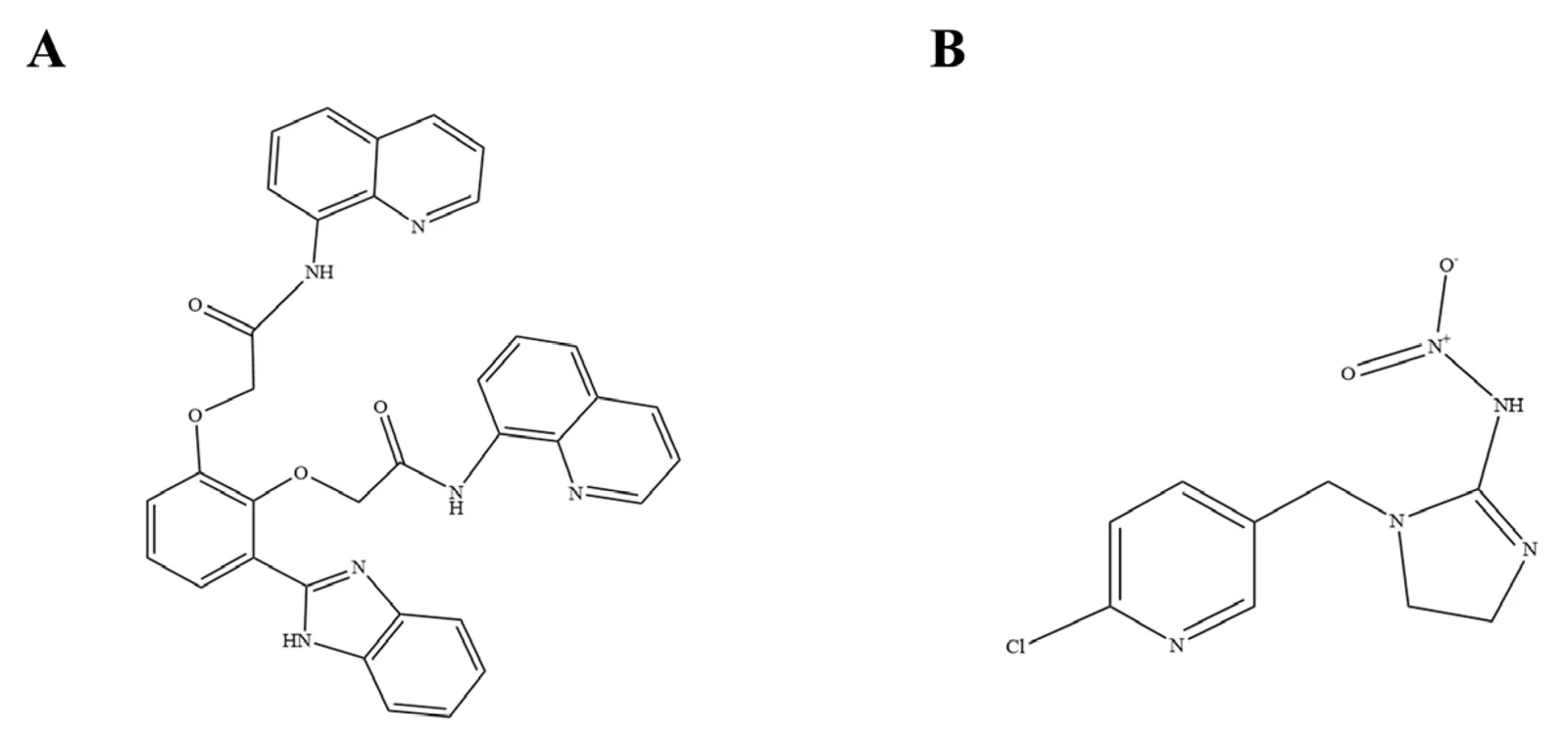
Structure of (**A**) **DQBM-B** and (**B**) IMI.

Quinoline-benzimidazole is a heterocyclic compound with a fused aromatic structure, which possesses both aromaticity and heterocyclic characteristics. In the structure of the **DQBM-B** probe (Figure 1A), the quinoline ring and the benzimidazole moiety collectively provide an extended π-conjugated surface that can engage in π–π stacking with the pyridine ring of IMI (Figure 1B), as well as intermolecular hydrogen bonding (N–H⋯N; N–H⋯O), thereby promoting the formation of a stable probe–IMI complex and realizing the detection of IMI.

In our previous work [22], the **DQBM-B** probe was developed for the selective recognition of Co^2+^ through a photoinduced electron transfer (PET) mechanism, wherein the benzimidazole N–H and quinoline nitrogen atoms serve as coordination sites for metal-ion binding. Notably, the quinoline–benzimidazole scaffold of **DQBM-B** also presents an extended π-conjugated aromatic surface and multiple hydrogen-bonding motifs, which endow the probe with the potential to interact with neutral organic molecules through non-covalent forces. IMI possesses a pyridine ring and nitroguanidine/2-chloropyridine moieties that are structurally complementary to the probe’s aromatic framework. Therefore, we hypothesized that **DQBM-B** could be repurposed for IMI detection by leveraging π–π stacking and hydrogen-bonding interactions to enrich IMI at the probe aggregate surface, thereby amplifying the inner filter effect (IFE) between the probe’s excitation band and IMI’s absorption band. This work thus represents an extension of the probe’s utility from metal-ion sensing to pesticide detection through a fundamentally different recognition mechanism.

We hypothesize that although the spectral overlap between the excitation spectrum of the probe and the absorption spectrum of IMI might be moderate in the monomeric state, the aggregated state could achieve local micro-enrichment of IMI via intermolecular hydrogen bonding and π–π interactions. This enrichment would increase the local concentration of the target analyte, which in turn amplifies the IFE between the probe and IMI. Consequently, a significant fluorescence quenching response is expected, realizing the highly sensitive detection of IMI.

Accordingly, this work systematically investigates the detection performance and sensing mechanism of the **DQBM-B** probe toward IMI. The main contributions of this study are as follows: (i) a metal-free fluorescent probe based on quinoline-benzimidazole is developed for IMI detection, eliminating the dependence on metal ions; (ii) the aggregation state of the probe is rationally regulated to construct a local enrichment interface for IMI, amplifying the IFE-based signal transduction; (iii) the sensing mechanism is elucidated through a combination of spectral analysis, fluorescence lifetime measurements, and DFT calculations, revealing the synergistic roles of hydrogen bonding, π–π stacking, and IFE; (iv) the matrix compatibility of the probe is evaluated in cucumber extracts as a proof-of-concept study, demonstrating the feasibility of the aggregation-regulated IFE strategy for neonicotinoid pesticide detection.

## 2. Results and Discussion

### 2.1. Feasibility Study on the Detection of IMI with **DQBM-B** Probe

**Figure 2:**
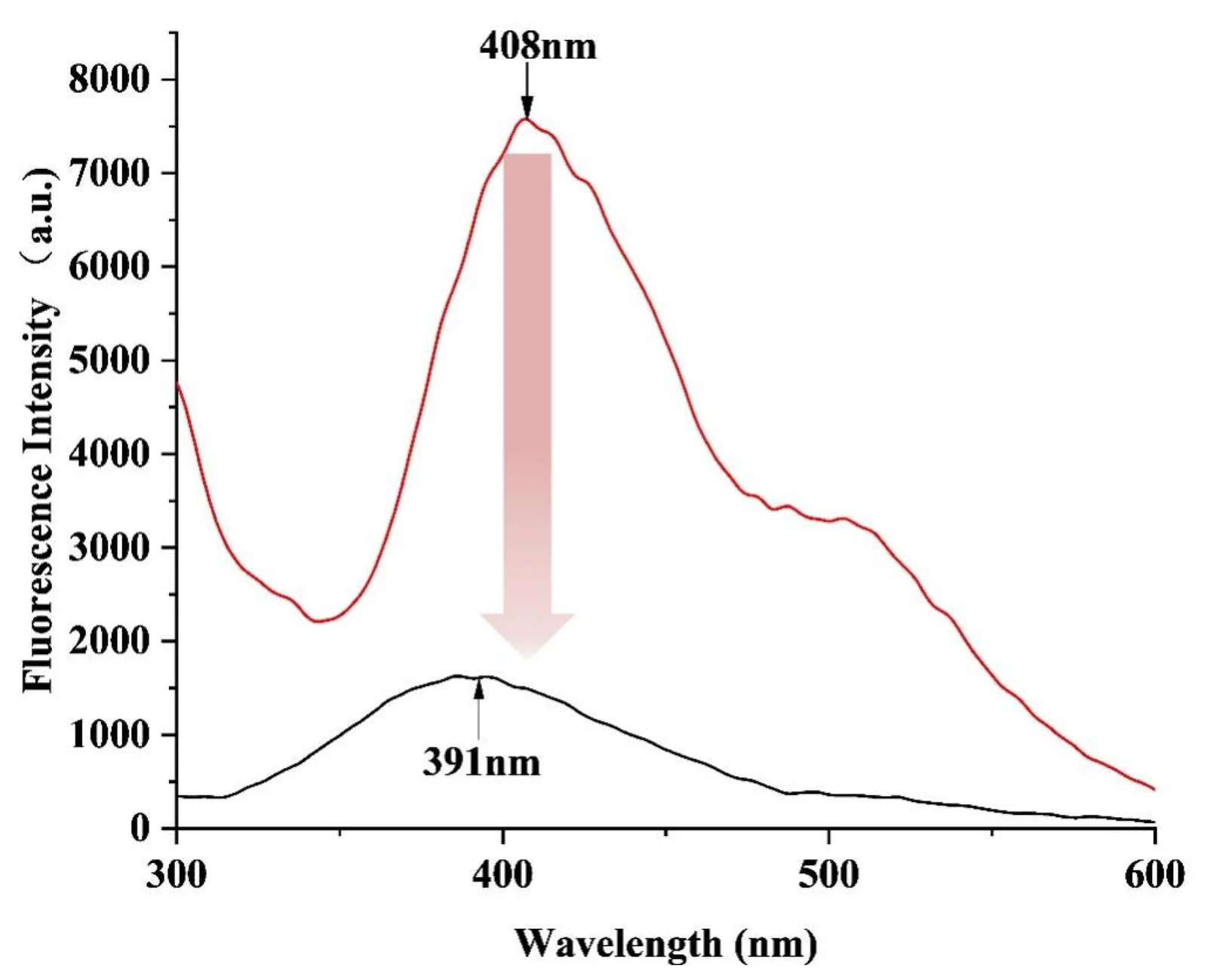
Fluorescence emission spectra of probe **DQBM-B** in the absence (the red curve) and presence (the black curve) of IMI (λ_ex_ = 274 nm). The red arrow indicates the blue-shift of the emission peak from 408 nm to 391 nm upon the addition of IMI, accompanied by the decrease in fluorescence intensity.

In the feasibility experiments, IMI solution was added dropwise to the probe solution successively, and the emission spectra after each addition were recorded. As can be seen from Figure 2, the fluorescence intensity of the probe decreased accompanied by a blue shift of the emission peak from 408 nm to 391 nm upon the addition of IMI. This spectral change can be rationalized by two synergistic physicochemical effects. First, the binding of IMI to the surface of the less polar **DQBM-B** aggregates through hydrogen bonding and π–π stacking interactions decreases the local polarity of the probe microenvironment. It is well documented that a fluorophore residing in a less polar, more hydrophobic environment emits at shorter wavelengths due to reduced solvent relaxation and consequent weaker stabilization of the excited state [23]. Second, the noncovalent interactions between **DQBM-B** and IMI restrict the intramolecular rotation of the probe, leading to conformational rigidification. Such supramolecular conformational confinement can alter the excited-state energy landscape and has been associated with emission spectral shifts in supramolecular assemblies [24]. Collectively, these results indicate that the probe undergoes both microenvironmental and conformational changes upon IMI binding.

### 2.2. Spectral Characteristics of **DQBM-B** Monomers and Aggregates

**Figure 3:**
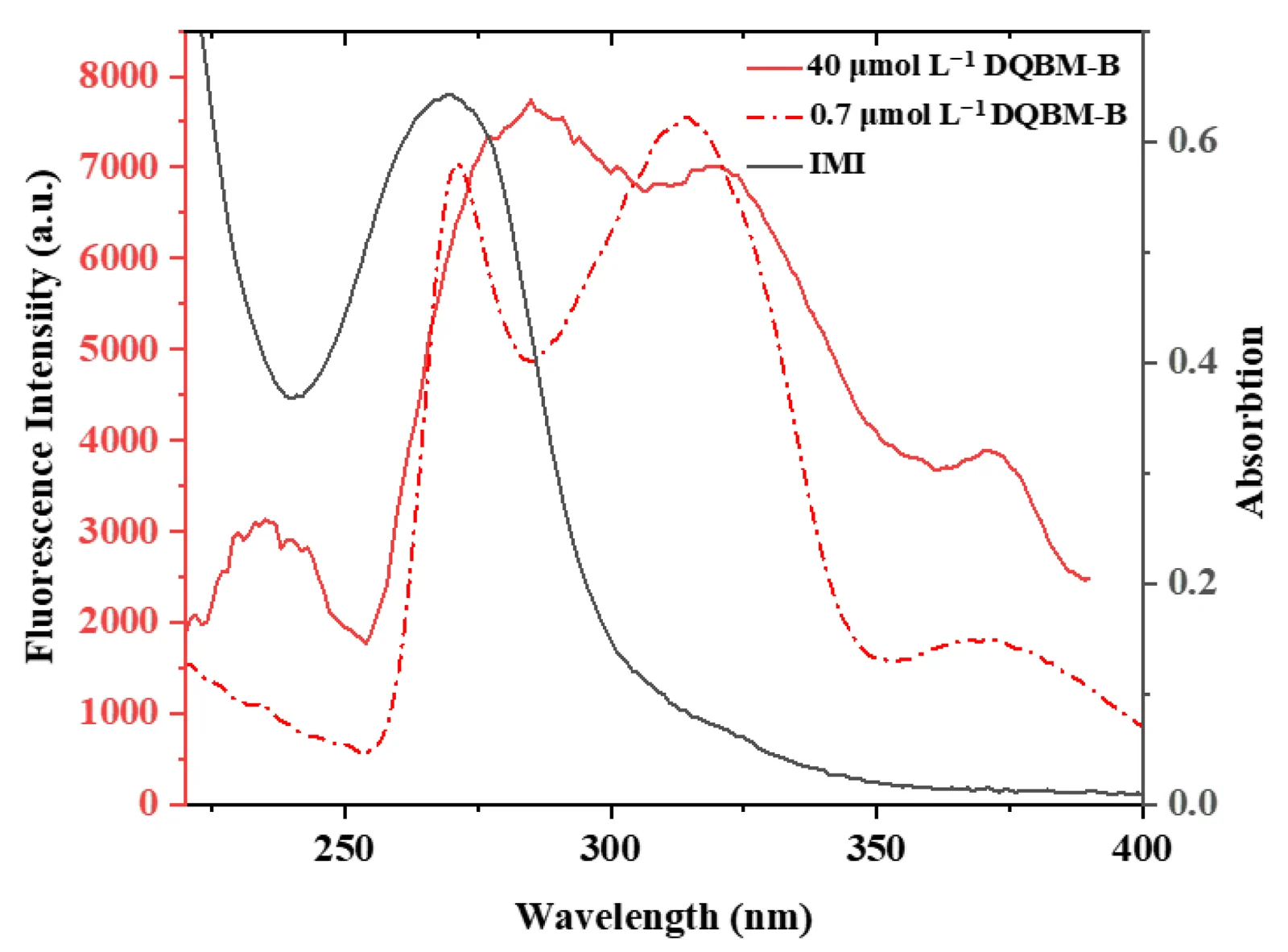
Fluorescence excitation spectra of probe **DQBM-B** at different concentrations (0.7 μmol L^−1^, 40 μmol L^−1^) (λ_em_ = 416 nm) and UV–vis absorption spectrum of IMI.

To clarify the influence of probe concentration on its spectral properties, the excitation spectra of two typical systems—low concentration (0.7 μmol L^−1^, monomeric state) and high concentration (40 μmol L^−1^, aggregated state)—were compared, along with their spectral overlap with the absorption spectrum of IMI, so as to analyze the spectral matching characteristics between the probe and the analyte under different aggregation states. As shown in Figure 3, in the monomer system (0.7 μmol L^−1^), the probe was highly dispersed as monomers, and its excitation spectrum exhibited sharp characteristic peaks with narrow full width at half maximum. The excitation spectrum of **DQBM-B** showed a large overlapping area with the absorption spectrum of IMI, indicating high spectral matching.

With a significant increase in probe concentration, the intermolecular distance was greatly reduced, and probe molecules self-assembled into stable aggregates through π–π stacking and intermolecular hydrogen bonding. Significantly affected by intermolecular interactions, the excitation spectrum was obviously broadened, the peak shape became smoother, and a distinct red shift was observed. Due to the red shift of the excitation spectrum, the overlapping area between the excitation spectrum of the probe and the absorption peak of IMI was relatively decreased. However, as demonstrated in Section 2.3, the aggregation state simultaneously creates a local enrichment interface that recruits IMI to the aggregate surface, thereby compensating for the reduced spectral overlap and ultimately enhancing the overall quenching efficiency. It is worth noting that the formation of such aggregates is associated with aggregation-caused quenching (ACQ), which arises from the strong intermolecular π–π stacking between probe molecules.

### 2.3. Optimization of Working Concentration

To determine the optimal working conditions of probe **DQBM-B** for IMI detection, a stock solution of the probe (700 μmol L^−1^ in DMF) was serially diluted with DMF to obtain working solutions at concentrations ranging from 0.7 to 700 μmol L^−1^. The effect of probe concentration on fluorescence quenching efficiency was then systematically investigated. The fluorescence quenching efficiency (η) was calculated as the ratio of the fluorescence change to the original intensity, according to the following Equation (1) [25]:

(1) $$\eta =\;\frac{F_{0}-F}{F_{0}}\times 100\%$$

where *F*_0_ and *F* are the fluorescence intensities of probe **DQBM-B** measured at the optimal emission wavelength (λ_em_ = 416 nm) in the absence and presence of IMI, respectively.

**Figure 4:**
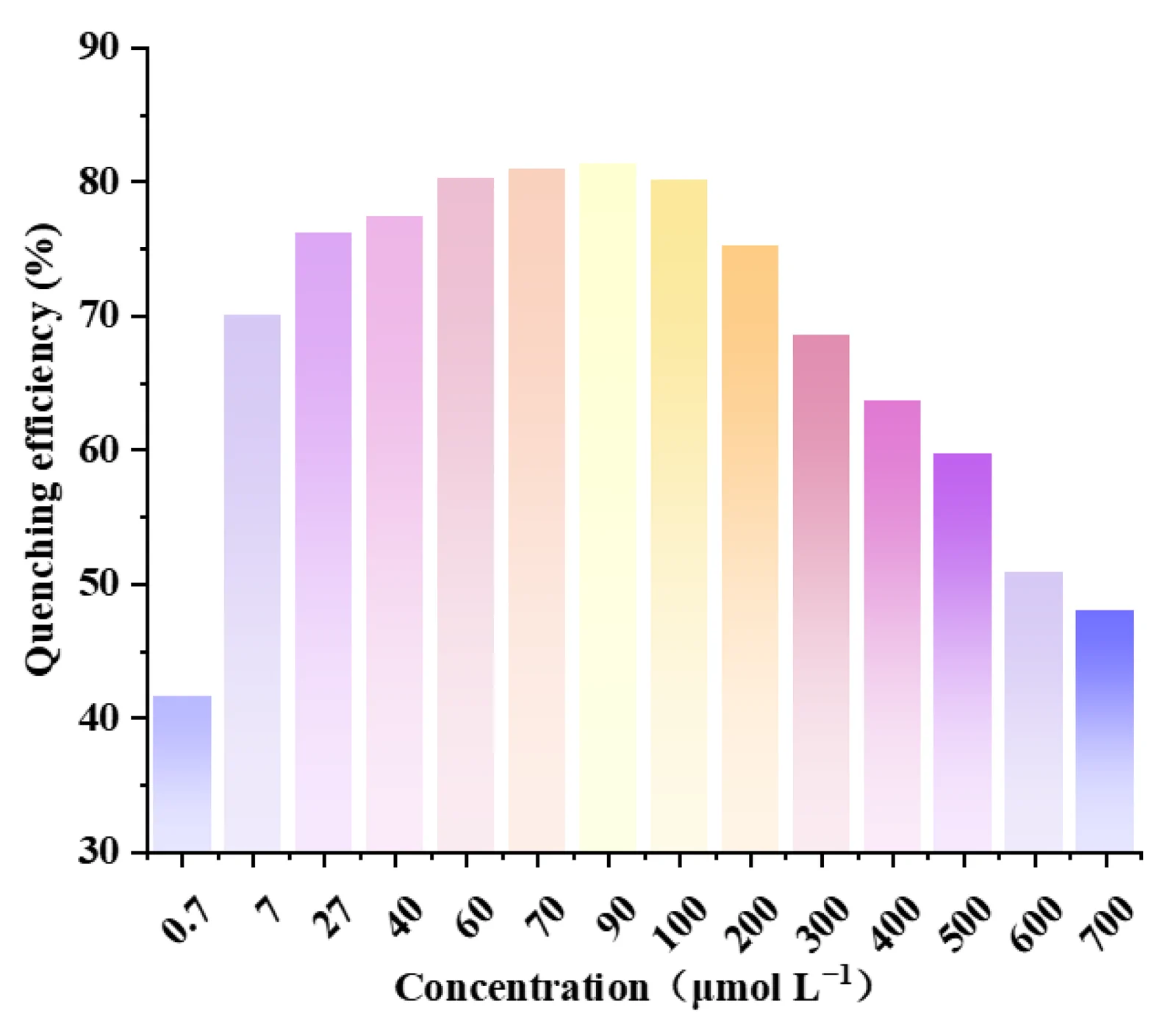
Quenching efficiency of **DQBM-B** at different concentrations toward IMI (λ_ex_ = 274 nm, λ_em_ = 416 nm).

The experimental results (Figure 4) showed that the quenching efficiency first increased and then decreased with the increase in probe concentration. The quenching efficiency was only 42% at 0.7 μmol L^−1^, significantly increased to 70% at 7 μmol L^−1^, reached 76% at 27 μmol L^−1^, and 77% at 40 μmol L^−1^, and stabilized at a plateau of 80–81% in the range of 60–90 μmol L^−1^. Although, the quenching efficiency is marginally higher at 60–90 μmol L^−1^, a concentration of 40 μmol L^−1^ was selected as the optimal working concentration for the following reasons. First, 40 μmol L^−1^ lies at the critical transition region from low to high aggregation, where moderate aggregation is conducive to the formation of an efficient enrichment interface without inducing excessive ACQ. Second, the background fluorescence signal remains moderate at this concentration, facilitating signal observation and subsequent optimization. In contrast, when the concentration exceeded 100 μmol L^−1^, the quenching efficiency began to decrease and dropped to 69% at 300 μmol L^−1^, likely because excessive aggregation reduces the background signal and thus diminishes the observable quenching response.

Notably, the concentration analysis results seem to contradict the spectral overlap (Figure 3). According to the spectral analysis in Section 2.2, the overlapping area between the excitation spectrum of the monomeric probe (0.7 μmol L^−1^) and the absorption spectrum of IMI is larger than that of the aggregated state (40 μmol L^−1^). If analyzed only based on IFE or FRET, the monomeric state should present a higher quenching efficiency. However, the concentration experimental results are completely opposite, indicating that factors other than spectral overlap affect the sensing process.

In-depth analysis based on the results suggests that the higher quenching efficiency of aggregates may result from the interaction between probe aggregates and IMI. Therefore, dynamic light scattering (DLS) measurements were performed on the probe monomers, probe aggregates, and the complex formed after adding IMI to the probe aggregates.

**Figure 5:**
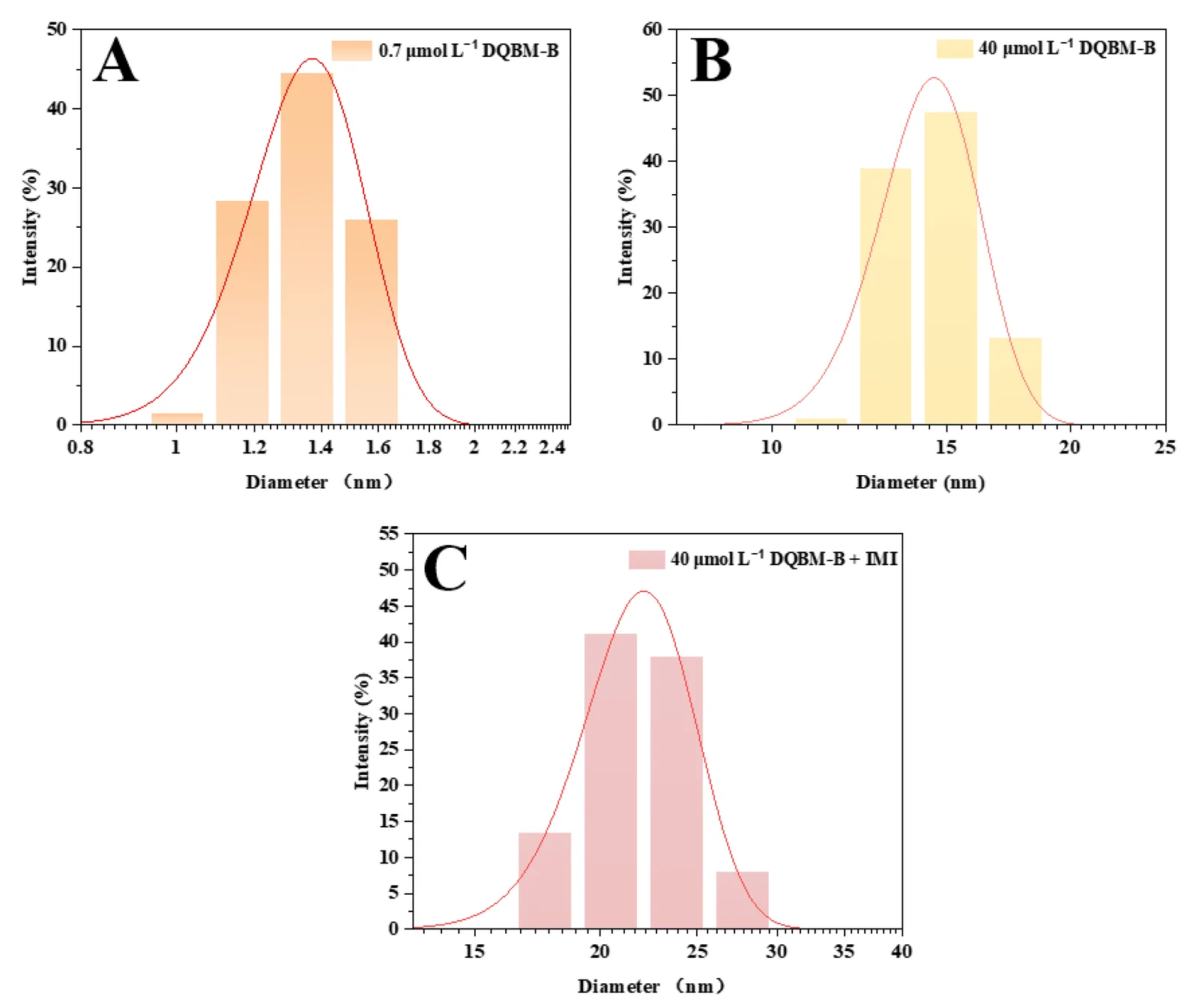
Dynamic Light Scattering (DLS) Particle Size Distribution of **DQBM-B** Under Different Conditions: (**A**) 0.7 μmol L^−1^ **DQBM-B** monomer dispersion; (**B**) 40 μmol L^−1^ **DQBM-B** aggregates dispersion; (**C**) particle size distribution of 40 μmol L^−1^ **DQBM-B** after adding IMI.

The probe **DQBM-B** itself exhibits ACQ characteristics, confirming that its molecular structure is prone to strong intermolecular π–π stacking. In the low-concentration monomeric state, the probe and IMI are uniformly dispersed in the solution with relatively random interactions, and the probe monomer molecules are wrapped by solvent molecules, which is not conducive to binding with IMI. The DLS data further reveal the dispersion characteristics of the probe in this state: the hydrodynamic diameter of the monomeric state (0.7 μmol L^−1^) is 1.35 nm in Figure 5A, which is close to the monomer size of porphyrin compounds (<1 nm) [26] and perylene diimide dimers (2–3 nm) [17], indicating that the probe is dispersed in the solution as single molecules. Since the probe concentration is higher than that of IMI, the excitation light can directly excite most unobstructed probe molecules, resulting in weak IFE and low quenching efficiency. When the probe concentration rises above the critical value, ordered aggregates are formed driven by intermolecular π–π stacking [27,28,29]. These π-stacked arrangements are further stabilized by intermolecular hydrogen bonding involving the amide N–-H and benzimidazole N–H donors, which collectively exclude solvent molecules from the interstitial spaces between probe molecules. The DLS data show that the particle size of the aggregated state (40 μmol L^−1^) increases to 15.18 nm (Figure 5B).

The feasibility experiments (Figure 2) revealed that the emission peak of the probe undergoes a certain blue shift after the addition of IMI. Given that IMI also has an aromatic ring structure, this spectral change indicates the occurrence of interactions between IMI and probe **DQBM-B**. The DLS data further confirm the existence of this interaction. After adding IMI, the particle size of the aggregates increases from 15.18 nm to 20.53 nm (Figure 5C). The increase in particle size may originate from the intermolecular hydrogen bonding and π–π stacking interaction between the probe and IMI, leading to the formation of a “probe–IMI” composite structure. The probe aggregates enrich IMI molecules at the aggregate surface through π–π stacking and hydrogen bonding, resulting in a local concentration of IMI near the probe that is much higher than its bulk concentration. At this time, the excitation light must first penetrate this “enrichment layer” of high-concentration IMI before reaching the probe, thus being largely absorbed, which significantly enhances the IFE. Therefore, although the overlapping area between the excitation spectrum of the aggregates and the absorption spectrum of IMI is reduced, the local high-concentration effect improves the actual absorption probability of the excitation light, ultimately leading to a higher quenching efficiency of the aggregated state than that of the monomeric state. In essence, the aggregation state transforms the probe from a dispersed fluorophore into a supramolecular scaffold that concentrates the analyte at its surface, thereby converting a weak IFE (in the monomeric state) into an efficient quenching response.

The concentration of 40 μmol L^−1^ is at the critical region of the transition from low aggregation to high aggregation. On the one hand, moderate aggregation is conducive to the formation of an efficient enrichment interface, thereby enhancing the IFE; on the other hand, excessive aggregation may lead to an overly strong ACQ effect, resulting in an excessively low background fluorescence, which is not conducive to signal observation. In addition, the background fluorescence signal of the probe is moderate at this concentration, facilitating subsequent optimization. Therefore, 40 μmol L^−1^ was selected as the experimental concentration for the follow-up studies in this work.

### 2.4. Optimization of Excitation Wavelength

**Figure 6:**
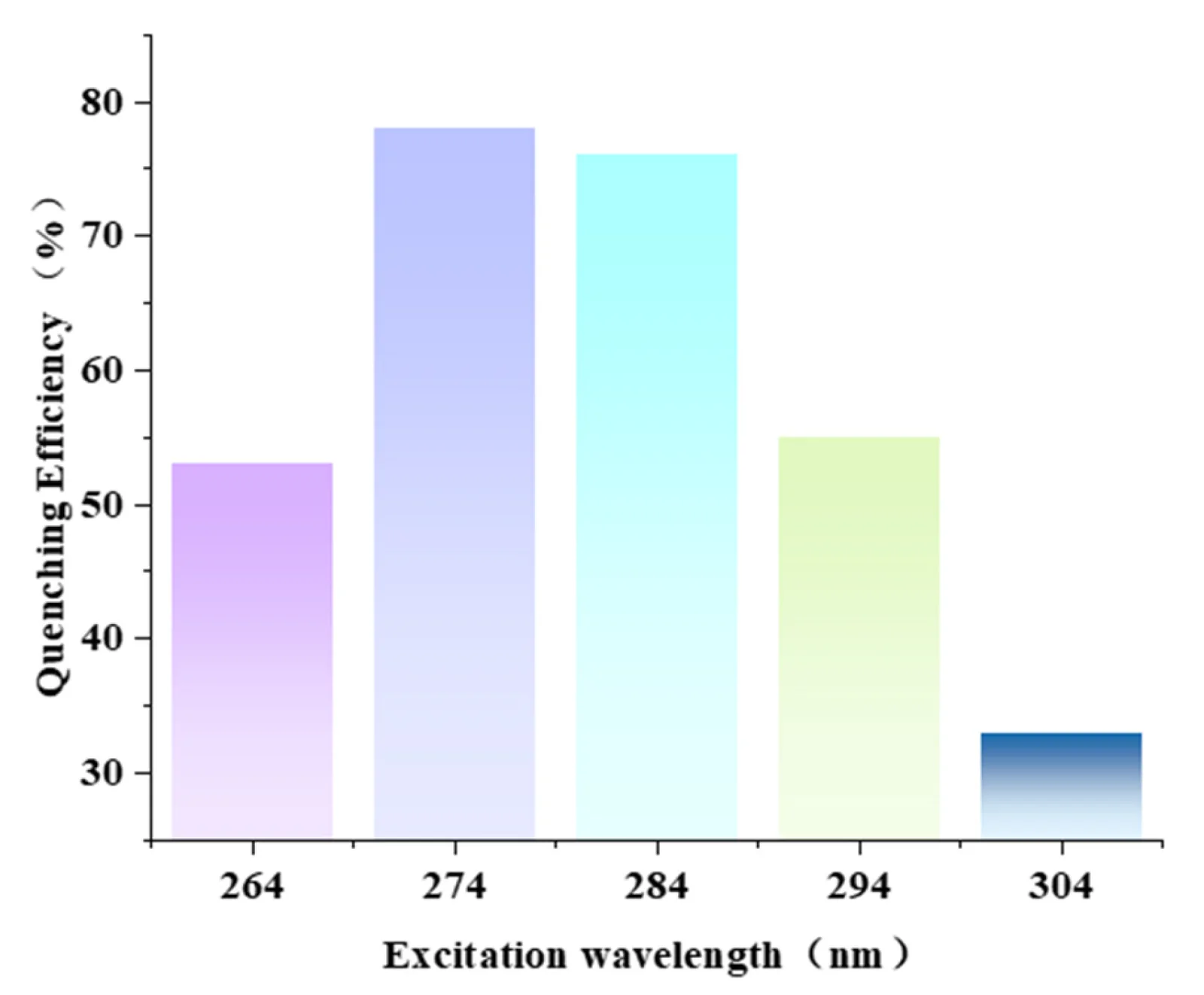
Quenching efficiency of **DQBM-B** toward IMI at different excitation wavelengths (λ_em_ = 416 nm).

To clarify the optimal excitation wavelength of probe **DQBM-B** for IMI detection, based on the IFE detection mechanism, this study conducted excitation wavelength optimization experiments in the wavelength range of 264–304 nm to investigate the effect of different excitation wavelengths on the fluorescence quenching efficiency. The experimental results are shown in Figure 6.

It can be seen from the figure that the excitation wavelength has a significant regulatory effect on the quenching efficiency of probe **DQBM-B** for IMI detection, showing an overall trend of “first increasing and then decreasing”: when the excitation wavelength is 264 nm, the quenching efficiency is 53%; as the excitation wavelength gradually increases to 274 nm, the quenching efficiency increases sharply to 78%, reaching the maximum value within the experimental range; continuing to increase the excitation wavelength to 284 nm, the quenching efficiency decreases slightly to 76%; when the excitation wavelength increases to 294 nm, the quenching efficiency drops to 55%, and further decreases to 33% at 304 nm.

This experimental phenomenon is consistent with the conclusion reported by Trang TT et al. [16]. Their study confirmed through the fluorescence quenching experiment of rhodamine B by triangular silver nanodisks that in the sensing system dominated by the IFE mechanism, the quenching efficiency is directly related to the matching degree of the excitation wavelength.

In summary, the present study reveals that the aggregation state of **DQBM-B** is essential for its sensing performance toward IMI. The probe self-assembles into nanoscale aggregates driven by π–π stacking and intermolecular hydrogen bonding, which creates a local enrichment interface that concentrates IMI at the aggregate surface. This enrichment layer acts as a physical filter, significantly amplifying the inner filter effect despite the reduced spectral overlap in the aggregated state compared to the monomer. Therefore, although the optimal excitation wavelength of probe **DQBM-B** itself is about 284 nm, based on the core requirements of the IFE detection mechanism and combined with the absorption spectral characteristics of IMI, this study determined 274 nm as the optimal excitation wavelength of probe **DQBM-B** for IMI detection. Under this condition, the absorption efficiency of IMI for the excitation light reaches the maximum, and the IFE effect is fully activated, which can provide a guarantee for the highly sensitive detection of IMI.

### 2.5. Fluorescence Titration

Different concentrations of IMI were added dropwise to the 40 μmol L^−1^ probe **DQBM-B** solution, and the change in fluorescence intensity of the system was monitored under an excitation wavelength of 274 nm to explore the response characteristics of the probe to IMI.

**Figure 7:**
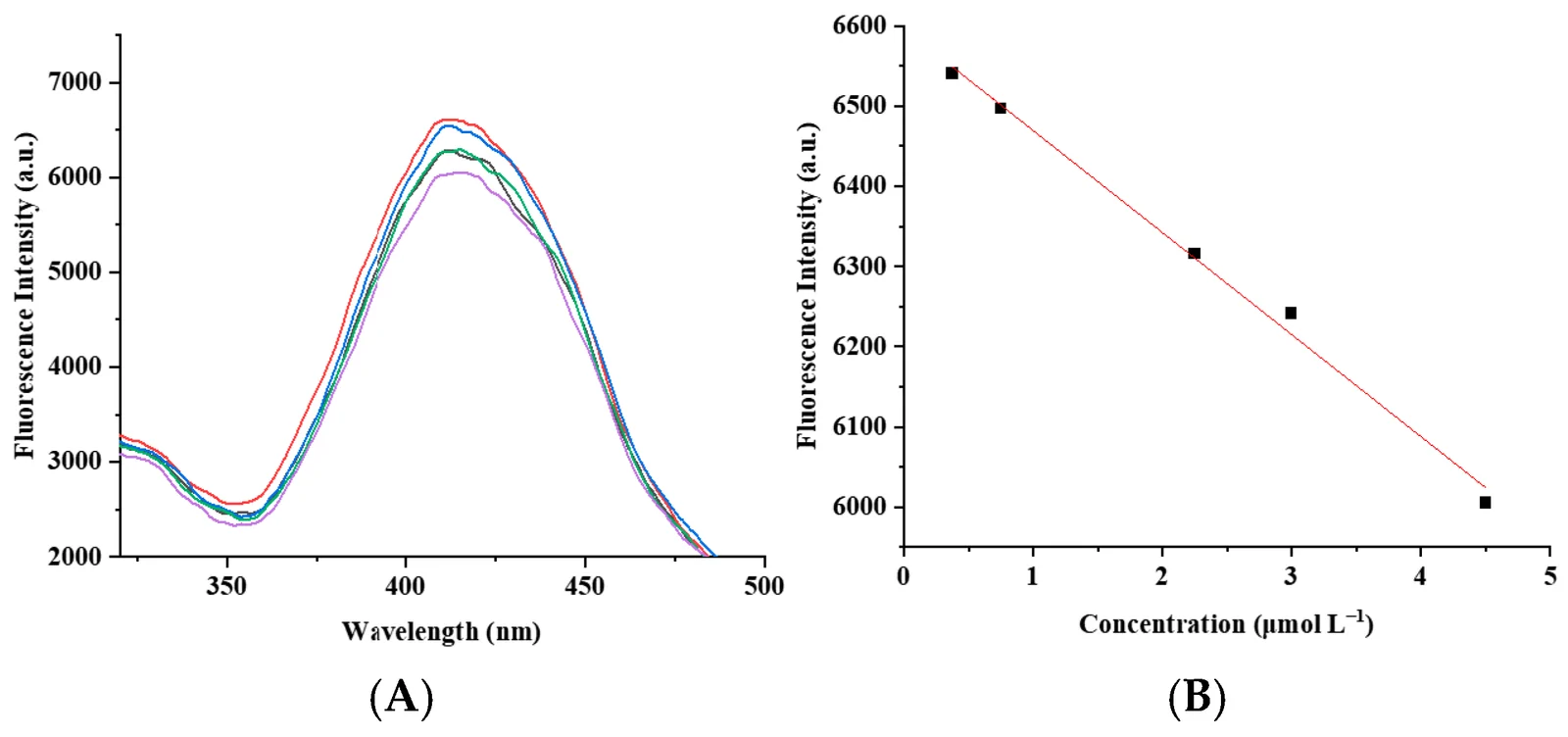
(**A**) Fluorescence spectra of probe **DQBM-B** after adding different concentrations of IMI (λ_ex_ = 274 nm). The different-colored lines represent fluorescence emission spectra corresponding to IMI concentrations of 0.375, 0.75, 2.25, 3.0 and 4.5 μmol L^−1^ in descending order. (**B**) Fluorescence intensity of probe **DQBM-B** after adding different concentrations of IMI (λ_ex_ = 274 nm, λ_em_ = 416 nm). The square symbols are the maximum fluorescence intensity values obtained from panel A, and the solid red line is the linear fitting curve.

The experimental results showed (Figure 7B) that with the increase in IMI concentration, the fluorescence intensity of probe **DQBM-B** presented a linear decreasing trend over the concentration range of 0.375–4.5 μmol L^−1^. Linear fitting yielded the equation y = −127.32x + 6596.82 (where y is the fluorescence intensity in a.u. and x is the IMI concentration in μmol L^−1^), with a correlation coefficient R^2^ = 0.9936, indicating a good linear correlation between the fluorescence intensity and IMI concentration. The limit of detection (LOD) of **DQBM-B** for IMI was calculated as 0.75 μmol L^−1^ according to Equation (2).

It should be explicitly noted that the LOD of 0.75 μmol L^−1^ (corresponding to approximately 0.19 mg L^−1^ or 190 ppb in solution) is substantially higher than the regulatory maximum residue limits (MRLs) for IMI in cucumber (0.5 mg kg^−1^ in the EU; 1 mg kg^−1^ by Codex) and in drinking water (typically ≤0.1 μg L^−1^). This sensitivity gap arises because the present study is designed as a proof-of-concept investigation of a new recognition mechanism based on aggregation-regulated IFE, rather than as a regulatory-compliant analytical method. The LOD reported here reflects the intrinsic sensitivity of the probe in homogeneous solution under the current experimental conditions, without any pre-concentration or signal-amplification steps. Consequently, the current method does not yet meet the legal requirements for residue monitoring in food or environmental samples, which must be acknowledged as a limitation of this exploratory work. Future efforts will focus on enhancing sensitivity through strategies such as ratiometric design, solid-phase extraction, or nanomaterial-assisted signal amplification to bridge the gap toward MRL-compliant detection.

(2) $$LOD=\frac{3\sigma }{S}$$

(*σ*: standard deviation of the blank response; *S*: the slope of the standard curve).

### 2.6. Selectivity and Anti-Interference

**Figure 8:**
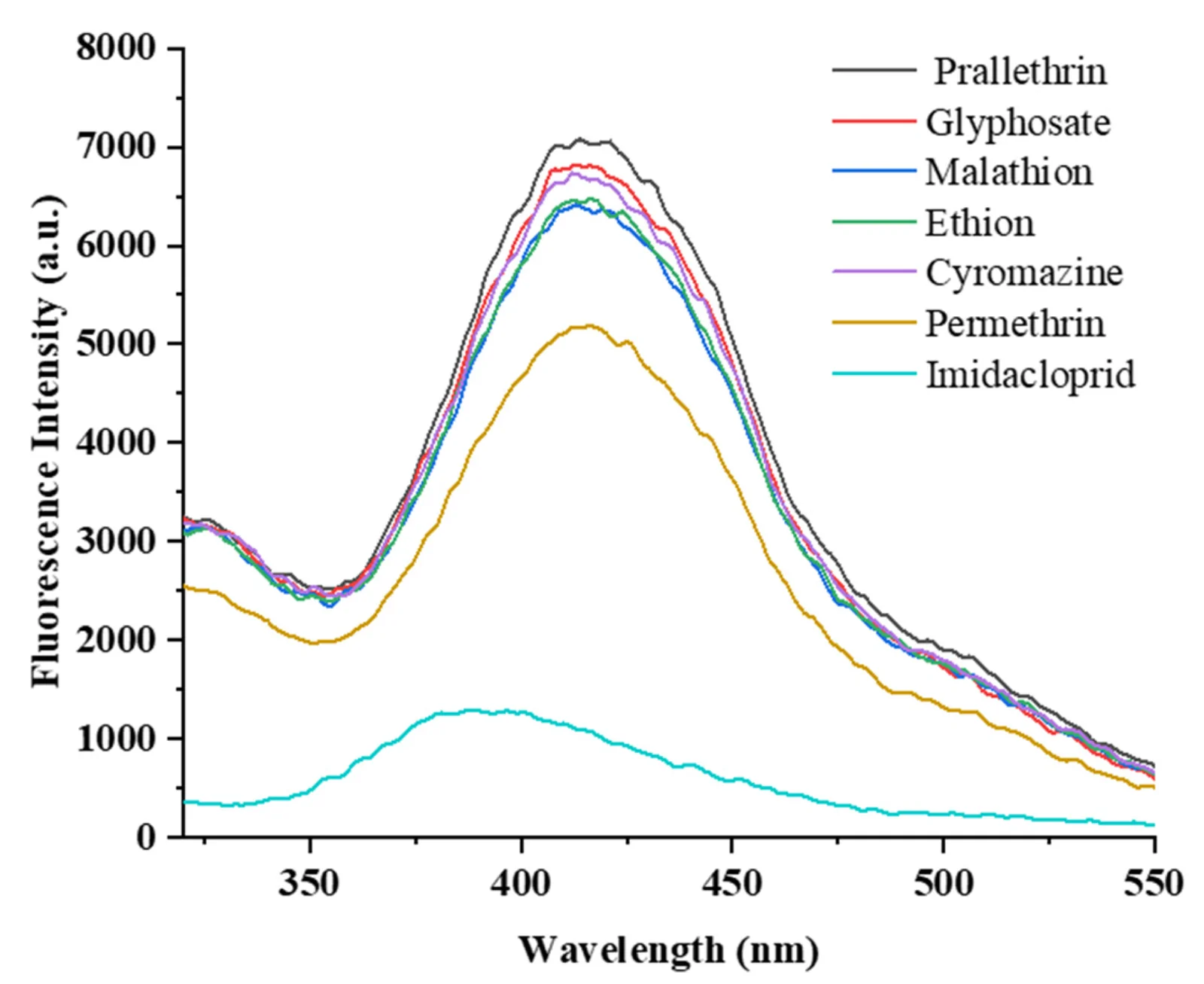
Fluorescence spectra of probe **DQBM-B** in the presence of different pesticides (λ_ex_ = 274 nm).

The fluorescence spectrum results in Figure 8 show that the probe **DQBM-B** itself exhibits a strong fluorescence signal in the wavelength range of 350–500 nm, with its emission peak at 416 nm and the maximum fluorescence intensity reaching 7365.17 (a.u.). When IMI was added to the probe solution, the fluorescence signal of the probe decreased significantly to a low level with a rapid response. Meanwhile, when 6 other common pesticides (glyphosate, prallethrin, malathion, ethion, cyromazine and permethrin) at the same concentration as IMI (7.5 μmol L^−1^) were separately added to the probe solution, the fluorescence intensity of the probe in the presence of glyphosate, prallethrin, malathion, ethion and cyromazine groups did not change significantly and remained basically consistent with the blank group containing only the probe.

Notably, permethrin caused a moderate decrease in the fluorescence signal. Quantitative analysis reveals that the standalone quenching effect of permethrin is 29.7%, and its relative interference percentage—calculated as [(*F*_0_ − *F*_perm_)/(*F*_0_ − *F*_IMI_)] × 100%—is 34.7%. This cross-interference may be attributed to high lipophilicity of permethrin (logP ≈ 6.5), which promotes non-specific hydrophobic adsorption onto the surface of the **DQBM-B** aggregates, rather than competitive binding at the specific IMI recognition sites. While this level of interference is not negligible when permethrin is present as the sole analyte, the following anti-interference experiments demonstrate that it does not compromise the probe’s practical selectivity for IMI in mixed systems.

**Figure 9:**
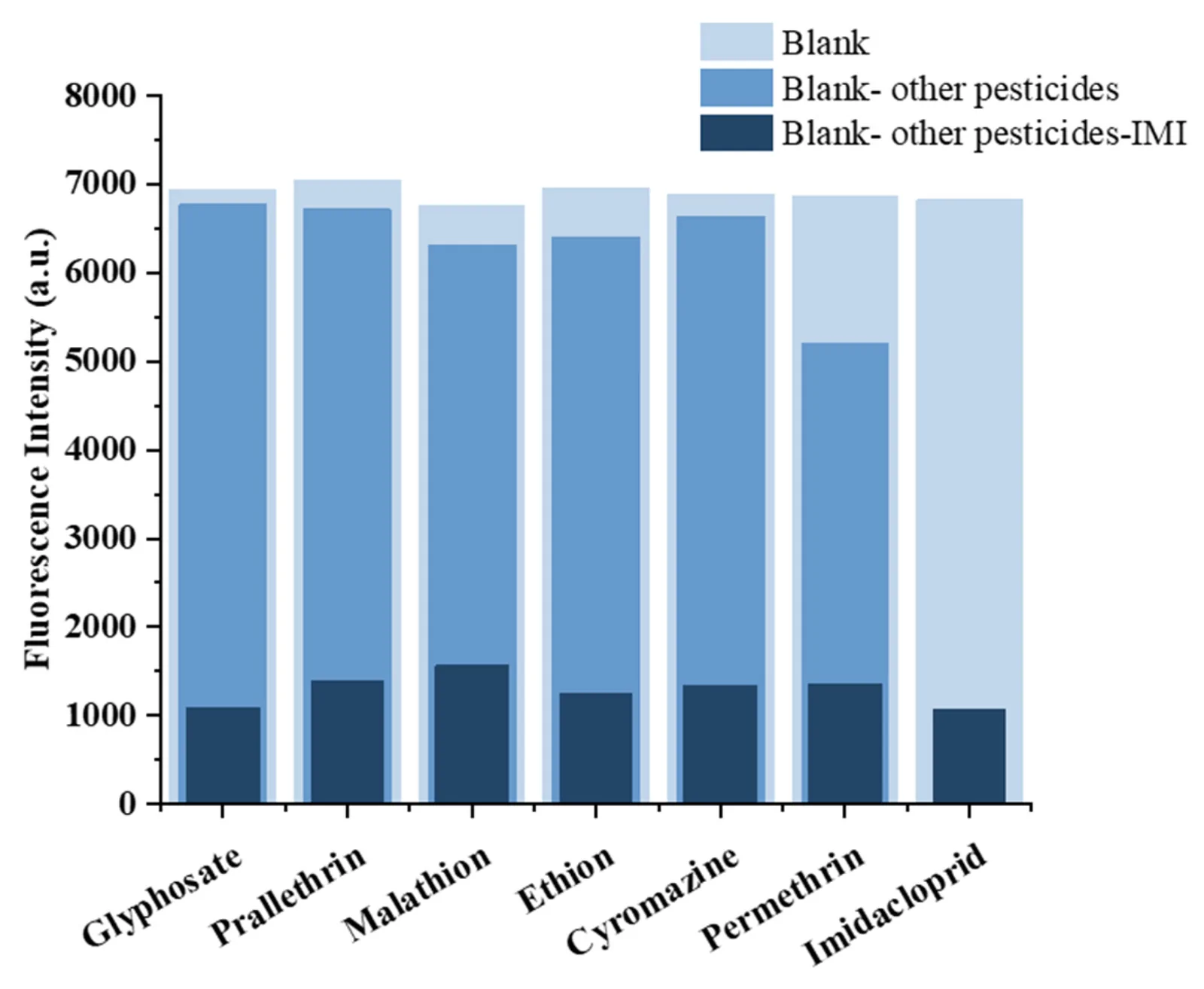
Fluorescence intensity of probe **DQBM-B** upon addition of IMI in the presence of different pesticides (λ_ex_ = 274 nm, λ_em_ = 416 nm).

An excellent fluorescent probe should still be able to reliably detect the target analyte in a system coexisting with various interfering substances. To verify this ability, an anti-interference test was performed on the probe **DQBM-B**. First, 10 μL of 7.5 μmol L^−1^ each competitive pesticide solution was added to the probe solution, and the fluorescence spectrum was recorded; then 10 μL of 7.5 μmol L^−1^ IMI solution was added, and the fluorescence spectrum was measured again. The experimental results show that even in the presence of the above competitive pesticides, the fluorescence intensity of the probe still decreased significantly after adding IMI (Figure 9).

Specifically, in the permethrin group, the fluorescence intensity dropped from 5196.67 a.u. (after permethrin pre-exposure) to 1358.24 a.u. (after subsequent IMI addition), which is comparable to the average endpoint of 1329.57 a.u. observed for the five non-interfering pesticides under identical conditions (difference < 2.2%). The decrease amplitude was basically the same as that in the absence of interfering pesticides, and the fluorescence intensity decreased to a level similar to that of the group treated with IMI alone. This indicates that the coexisting competitive pesticides did not affect the specific binding between the probe and IMI, nor did they interfere with the occurrence of the fluorescence quenching process. Notably, although permethrin alone causes a moderate quenching effect through non-specific hydrophobic adsorption (Figure 8), its presence does not impede the subsequent quenching by IMI (Figure 9), suggesting a non-competitive interference mechanism that does not block the binding sites essential for IMI recognition. This experiment demonstrates that the probe **DQBM-B** can accurately recognize the target analyte IMI even in complex multi-component systems, possessing good anti-interference ability.

### 2.7. Detection Mechanism

**Figure 10:**
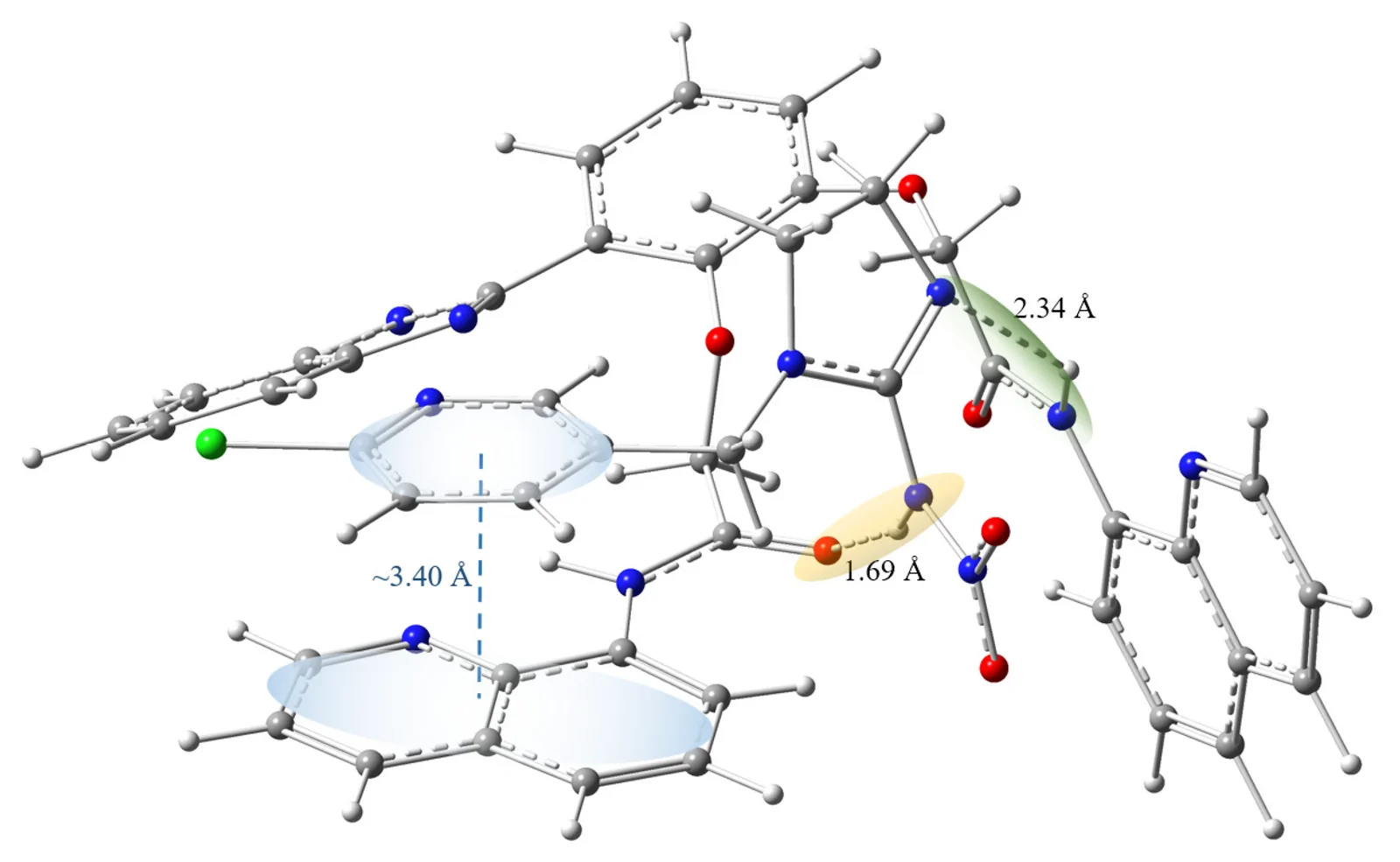
Schematic diagram of hydrogen bonding interactions and π–π stacking interactions between the **DQBM-B** and IMI. The gray, blue, red and white spheres represent carbon, nitrogen, oxygen and hydrogen atoms, respectively.

To better understand the binding mode between the **DQBM-B** probe and IMI, density functional theory (DFT) calculations were performed on free **DQBM-B**, free IMI, and the **DQBM-B**–IMI complex. It should be noted that these DFT optimizations were conducted for monomeric complexes in DMF implicit solvent, which reveal intrinsic noncovalent interaction patterns but do not fully represent the aggregated sensing environment. The DFT calculations reveal a thermodynamically favorable interaction between **DQBM-B** and IMI. As shown in the optimized complex structure (Figure 10), multiple noncovalent interactions collectively contribute to the formation of a stable probe–IMI complex. Specifically, an intermolecular hydrogen bond is formed between **DQBM-B** and IMI: the lengths of the N–H⋯N and N–H⋯O hydrogen bonds are 2.34 Å and 1.69 Å, respectively, confirming the existence of effective hydrogen bonding interactions. Meanwhile, the calculated plane distance between the quinoline aromatic ring of **DQBM-B** and the pyridine ring of IMI is ~3.44 Å, which falls within the typical range for π–π stacking interactions, demonstrating the presence of π–π interactions between the probe and IMI. These molecular interactions serve two distinct functions: (i) they form a stable ground-state complex, which contributes to static quenching by reducing the population of free emissive probe molecules; and (ii) they anchor IMI to the probe/aggregate surface, creating a local enrichment layer that maximizes the local concentration of the analyte. This enrichment is a prerequisite for the observed IFE, rather than a direct photophysical quenching pathway that alters the excited state of the fluorophore.

**Figure 11:**
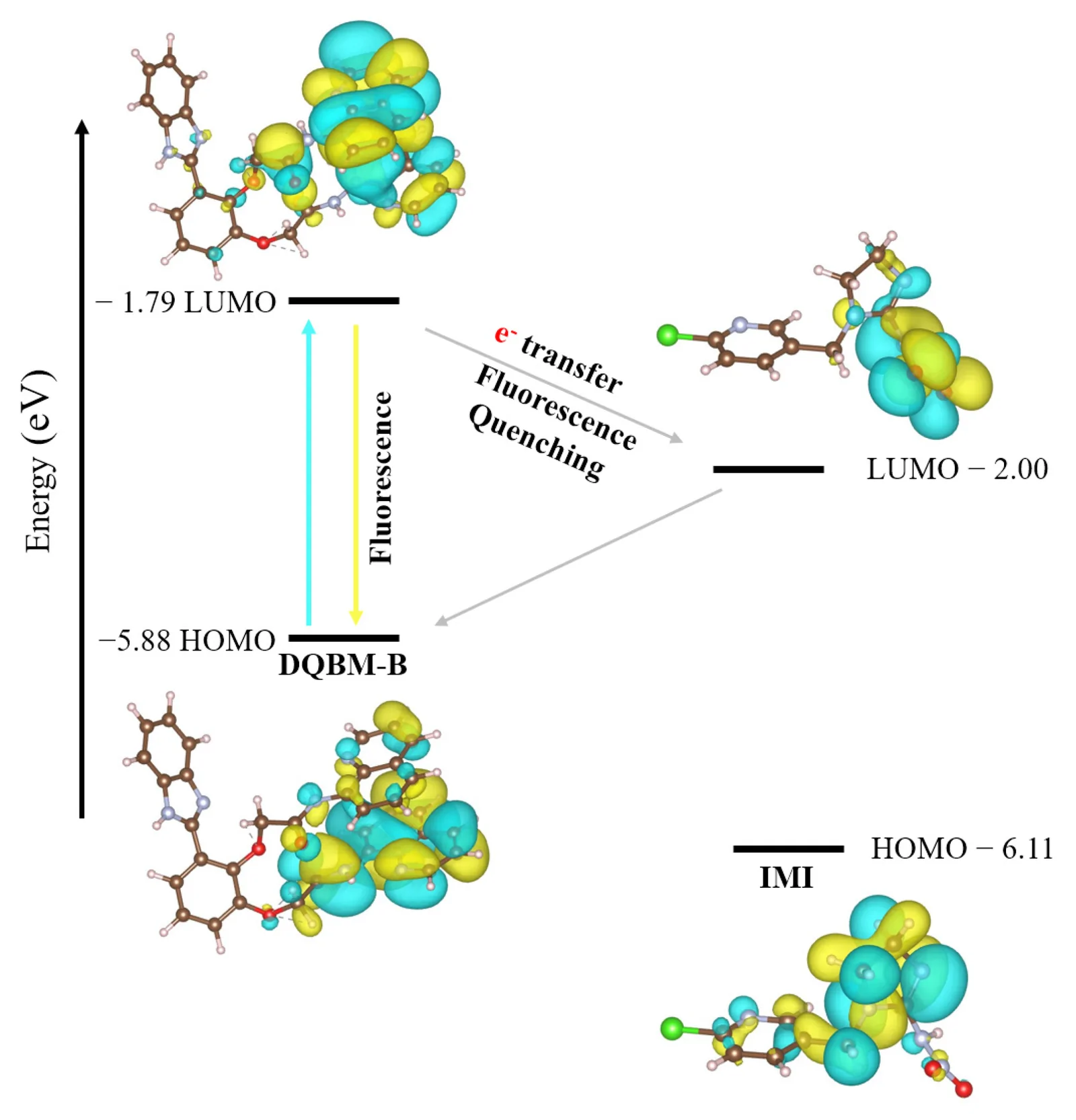
Calculated frontier molecular orbitals (HOMO and LUMO) and energy levels for free **DQBM-B** and the **DQBM-B**–IMI complex.

**Figure 12:**
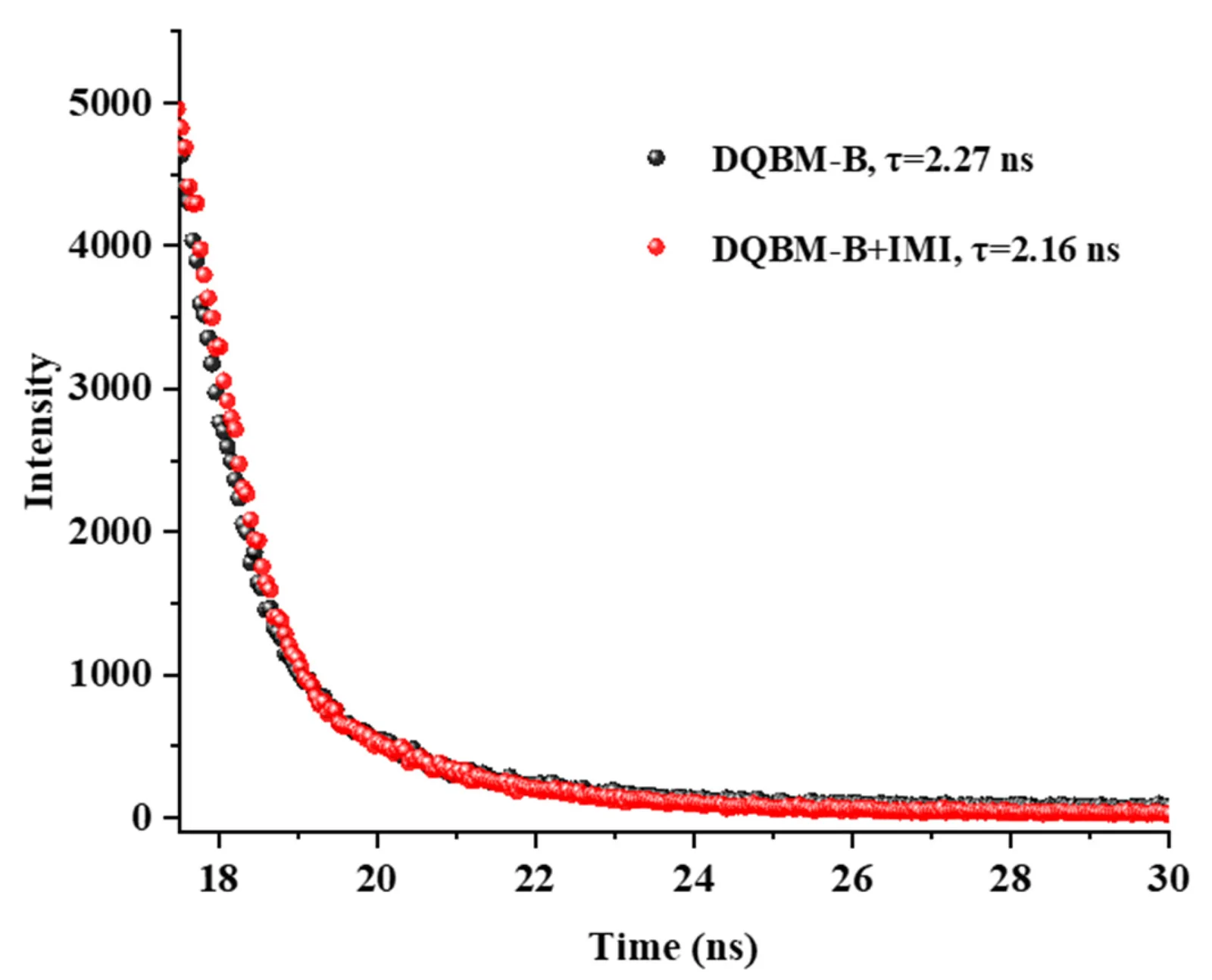
Fluorescence lifetimes of probe **DQBM-B** before and after the addition of IMI.

To clarify whether IMI binding alters the electronic structure of **DQBM-B**, the frontier molecular orbitals (HOMO and LUMO) were analyzed before and after complex formation. As shown in Figure 11, both the HOMO and LUMO remain predominantly localized on the **DQBM-B** moiety in the **DQBM-B**–IMI complex, with negligible contribution from IMI. Moreover, the HOMO–LUMO energy gap changes only marginally upon complexation. These results indicate that IMI binding does not significantly perturb the overall electronic structure of **DQBM-B**. Combined with the fluorescence lifetime measurements (Figure 12), which show only a marginal decrease upon IMI addition (2.27 ns → 2.16 ns), this definitively rules out PET, and other excited-state quenching mechanisms in the actual sensing system. Furthermore, the DFT calculations were performed on monomeric species in DMF implicit solvent and do not account for the electronic structure of the aggregated state.

To further investigate the fluorescence quenching mechanism of the **DQBM-B** probe toward IMI, the spectral properties of the probe and IMI were analyzed. The excitation spectrum of probe **DQBM-B** showed a significant overlap with the UV-Vis absorption spectrum of IMI (Figure 3), satisfying the basic conditions for the occurrence of either FRET or IFE. Therefore, it was preliminarily speculated that the fluorescence quenching between the two might originate from the FRET process or IFE.

The occurrence of IFE relies on the competitive absorption of excitation or emission light by the quencher, which only acts on the ground-state light absorption process and does not alter the excited-state lifetime of the fluorophore. In contrast, the quenching mechanism of FRET is usually accompanied by a significant decrease in the fluorescence lifetime of the donor. To distinguish between the above mechanisms, the fluorescence lifetime of probe **DQBM-B** was measured (Figure 12). The fluorescence lifetime data were fitted using an exponential model (the residual value ***χ*^2^** ≤ 1.2):

(3) $$R\left(t\right)=\sum_{i}e^{-\frac{t}{\tau_{i}}}$$

*R*(*t*): The fluorescence intensity at time t; *B_i_*: The pre-exponential factor of the *i*-th term at time *t*; *τ_i_*: The fluorescence lifetime of the *i*-th term.

The average fluorescence lifetime is calculated using the following formula:

(4) $$\tau_{av}=\frac{\sum \alpha_{i}\tau_{i}^{2}}{\sum \alpha_{i}\tau_{i}}$$

*α_i_*: The proportion of the lifetime *τ_i_*.

The results (Figure 12) showed that the fluorescence lifetime of probe **DQBM-B** alone was 2.27 ns, and only slightly decreased to 2.16 ns after the addition of IMI, with no significant difference. This indicated that the quenching process did not involve excited-state interactions, thus excluding mechanisms such as FRET or PET.

Combining the spectral overlap analysis, fluorescence lifetime measurements, and DFT calculations, the fluorescence quenching mechanism of probe **DQBM-B** toward IMI is attributed to the synergistic contribution of static quenching and the inner filter effect (IFE). Specifically:

(i) Static quenching: The DFT-optimized complex structure (Figure 10) reveals the formation of a stable ground-state complex via hydrogen bonding and π–π stacking, with a calculated binding energy of −14.3 kcal/mol. This reduces the population of free emissive probe molecules without altering the excited-state lifetime of the remaining unbound probes.
(ii) Inner filter effect (IFE): The same noncovalent interactions that form the ground-state complex also anchor IMI to the aggregate surface, creating a high-concentration absorption layer. This layer competitively absorbs excitation light (and/or reabsorbs emitted light) before it reaches the fluorophore, giving rise to the IFE. It is critical to clarify that the molecular recognition interactions (H-bonding and π–π stacking) serve to concentrate IMI at the probe surface, thereby maximizing the IFE; they do not directly alter the excited-state photophysics of **DQBM-B**. Both static quenching and IFE are ground-state phenomena that leave the excited-state lifetime of unquenched probe molecules essentially unchanged, which is fully consistent with the observed marginal decrease (2.27 ns → 2.16 ns) and definitively excludes dynamic mechanisms such as FRET or PET.

Consequently, efficient fluorescence quenching of **DQBM-B** is achieved, endowing the probe with sensing capability toward IMI.

### 2.8. Matrix Effect Evaluation in Cucumber Extract

To evaluate potential matrix interference from cucumber on the probe response, cucumber extracts were spiked with IMI at concentrations of 0.375, 2.25 and 4.50 μmol L^−1^, which fall within the linear range of the probe. As noted in Section 2.5, these spiking levels correspond to approximately 6860 mg kg^−1^ in the homogenized sample prior to dilution, far exceeding regulatory MRLs; therefore, this experiment is intended solely to assess matrix compatibility within the probe’s detectable range, not to simulate regulatory-level analysis.

The experimental results are listed in Table 2. The recoveries of IMI were in the range of 101–106%, with RSD values up to 8%. These results indicate that the cucumber matrix exerts negligible interference on the response of the **DQBM-B** probe within its detectable concentration range, supporting its potential for rapid screening purposes. Nevertheless, further optimization (e.g., signal amplification or pre-concentration) would be required to achieve MRL-compliant detection.

## 3. Materials and Methods

### 3.1. Reagents

2,2′-((3-(1H-benzo[d]imidazol2-yl)-1,2-phenylene) bis(oxy)) bis(N-(quinolin-8-yl) acetamide) (**DQBM-B**) was synthesized as reported previously [22]. Pesticides including prallethrin, permethrin, malathion, and ethion were purchased from Tammo Quality Inspection Technology Co., Ltd. (Shanghai, China). Glyphosate and cyromazine were obtained from Shanghai Macklin Biochemical Co., Ltd. (Shanghai, China). IMI was purchased from Shanghai Haoyuan Biomedical Technology Co., Ltd. (Shanghai, China). N,N-Dimethylformamide (DMF) of analytical grade was supplied by Tianjin Fuyu Fine Chemical Co., Ltd. (Tianjin, China).

### 3.2. Structural Characterizations

UV–vis absorption spectra were recorded on a UV-Vis spectrophotometer (UV1800, Shanghai Mapada Instruments Co., Ltd., Shanghai, China). Fluorescence spectra were measured using a Fluorescence Spectrometer (FP8550, Hitachi High-Technologies Trading Co., Ltd., Tokyo, Japan). Time-resolved fluorescence decay profiles were recorded on a Steady-State/Transient Fluorescence Spectrometer (FS1000, Edinburgh Instruments Ltd., Edinburgh, UK). Weighing was performed with an electronic analytical balance (AL140, Mettler-Toledo Instruments Co., Ltd., Greifensee, Switzerland). Sample mixing was carried out on a vortex mixer (MX-S, Dragon Laboratory Instruments (Beijing) Co., Ltd., Beijing, China). Particle size and zeta potential analyses were performed using a Zetasizer Pro (Malvern Panalytical Ltd., Malvern, UK).

Software. Quantum chemical calculations were performed using Gaussian 16 (Revision A.01, Gaussian, Inc., Wallingford, CT, USA) [30]. Electronic structure analyses were conducted using Multiwfn (ver. 3.8, http://sobereva.com/multiwfn) [31], and the resulting isosurfaces were visualized using VMD (ver. 1.9.3, https://www.ks.uiuc.edu/Research/vmd) [32]. UV–vis absorption spectra were recorded and processed using the manufacturer-supplied software of UV1800. Fluorescence emission spectra were recorded and processed using the manufacturer-supplied software of FP8550. Time-resolved fluorescence decay profiles were recorded and processed using Fluoracle (ver. 2.5, Edinburgh Instruments Ltd., Edinburgh, UK). Particle size and zeta potential data were analyzed using ZS Xplorer (ver. 3.1, Malvern Panalytical Ltd., Malvern, UK). Molecular structures were drawn using ChemDraw (ver. 22.2, PerkinElmer, Waltham, MA, USA), available online: https://www.perkinelmer.com (accessed on 19 August 2026). Statistical analyses and plotting were performed using OriginPro (ver. 2023b, OriginLab Corporation, Northampton, MA, USA), available online: https://www.originlab.com (accessed on 19 August 2026).

### 3.3. Theoretical Calculations

Density-functional theory (DFT) was carried out with Gaussian 16 [30]. Electronic structure calculations were carried out using the B3LYP functional [33] in conjunction with Grimme’s D3BJ dispersion correction [34]. The def2-SVP basis set [35] was employed to describe all atoms in geometry optimizations and frequency calculations. The B3LYP-D3BJ/def2-TZVP level was utilized for single-point energy evaluations. Implicit solvation was accounted for via the Solvation Model Based on Density (SMD) [36] in geometry optimizations and frequency calculations. DMF served as the solvent. Frontier orbital analyses were performed using Multiwfn (ver. 3.8) [31], and the resulting isosurfaces were visualized with VMD (ver. 1.9.3) [32].

### 3.4. Preparation of Pesticide Stock Solutions for Selectivity and Anti-Interference Studies

For the selectivity and anti-interference studies (Section 2.6), stock solutions (7.5 μmol L^−1^) of prallethrin, glyphosate, malathion, ethion, cyromazine, permethrin, and IMI were prepared by dissolving their respective standard products in DMF. Briefly, an accurately weighed amount of each pesticide standard was dissolved in DMF with sufficient sonication, then transferred to a volumetric flask and brought to volume with DMF. All stock solutions were stored at 4 °C in the dark. Unless otherwise stated, all experimental solutions had a total volume of 200 μL, composed of the **DQBM-B** probe stock solution and the pesticide stock solution. All solutions, whether spiked with target pesticides or not, were thoroughly mixed at room temperature before use.

### 3.5. Sample Preparation and Extraction Procedure for Matrix Effect Evaluation in Cucumber

The extraction solvent for this study was selected based on the physicochemical requirements of the **DQBM-B** probe. Preliminary experiments revealed that the probe is virtually insoluble in acetonitrile, ethanol, and methanol, precluding the formation of the ordered aggregates essential for the sensing mechanism. In contrast, DMF provides optimal probe solubility and supports the formation of well-defined aggregates (15.18 nm, Figure 5), which are critical for the local enrichment of IMI and the subsequent IFE-based signal transduction. While DMF is not the most environmentally friendly solvent and is not the standard choice for regulatory pesticide analysis (e.g., QuEChERS), it has been employed in pesticide residue extraction, particularly for lipid-rich matrices using DMF–hexane partitioning systems. We acknowledge that future work should explore greener solvent alternatives through probe structural modification or surfactant-assisted dispersion strategies.

For method-validation purposes, a recovery test was performed on homogenized cucumber samples [37]. Fresh cucumber (37 g) was homogenized, and 0.5 g of the homogenized sample was spiked with 3.43 mg of IMI. The spiked homogenate was briefly vortex-mixed to ensure homogeneous distribution and then immediately extracted with DMF under sonication for 10 min, followed by centrifugation at 10,000 rpm for 10 min. The supernatant was filtered through a 0.22 μm membrane filter and diluted with DMF to the required concentrations. Finally, cucumber samples containing 0.375 μmol L^−1^, 2.25 μmol L^−1^, and 4.50 μmol L^−1^ of IMI were obtained for the subsequent detection. Because the tissue had been mechanically disrupted prior to fortification, the cellular structures were no longer intact; thus, the recovery data reflect the efficiency of the extraction and detection protocol, rather than the kinetics of pesticide penetration across intact cell walls.

## 4. Conclusions

Based on the previously synthesized quinoline-benzimidazole derivative fluorescent probe **DQBM-B**, this work focused on investigating its detection performance and mechanism for the neonicotinoid pesticide IMI in the aggregated state. Experiments confirmed that the **DQBM-B** aggregates could successfully achieve selective recognition of IMI. Through systematic optimization of detection parameters, the optimal working concentration of the probe and the optimal excitation wavelength were determined, providing reliable conditions for subsequent detection experiments. Mechanistic studies revealed that the recognition of IMI by this probe aggregates was not attributed to a single mechanism. Rather, synergistic intermolecular hydrogen bonding and π–π stacking interactions enriched IMI at the aggregate surface, leading to fluorescence quenching of the probe via IFE.

Performance test results showed that under the optimal detection conditions, the LOD of this sensing system for IMI was 0.75 μmol L^−1^. Anti-interference experiments verified that the system still maintained a stable fluorescence response in complex environments coexisting with various common pesticides, demonstrating satisfactory anti-interference ability. As a proof-of-concept matrix compatibility study, the probe was applied to spiked cucumber extracts. Recoveries ranged from 101% to 106% with relative standard deviations below 10%, indicating that the matrix components did not significantly interfere with the IFE-based detection. It should be explicitly noted that the spiking levels used in this study were above regulatory MRLs, and the current LOD does not yet meet the requirements for practical regulatory-level screening. Future work will focus on enhancing the sensitivity—through structural modification of the fluorophore or signal amplification strategies—to bridge this gap.

In summary, this work expanded the detection application scope of the fluorescent probe **DQBM-B** from metal-ion sensing to pesticide detection via a fundamentally different mechanism, and provided a new metal-free, aggregation-regulated IFE strategy for the fluorescence detection of neonicotinoid pesticides. While the present study establishes the chemical feasibility of this approach, further optimization is required to achieve the sensitivity necessary for real-world agricultural monitoring.

## 5. Patents

This research supported Chinese patent application 202610878396.2 (Title: Application of a Quinoline-Benzimidazole Fluorescent Probe in the Detection of Neonicotinoid Insecticides).

## Figures and Tables

**Table 1:** Comparison of recently reported fluorescent sensors for imidacloprid detection.

Probe/Method	Technique	Linear Range	LOD	Sample Matrix	Metal-Free	Ref.
N-doped carbon dots	Fluorescence	0.1–50 μmol L^−1^	0.03 μmol L^−1^	Environmental water	N (Cu^2+^)	[9]
Zr(IV)-MOF	Fluorescence	0.01–10 μmol L^−1^	0.005 μmol L^−1^	Water, serum	N (Zr^4+^)	[10]
Tb-carbon QDs	Luminescence	0.05–50 ng mL^−1^	0.015 ng mL^−1^	Caneberries	N (Tb^3+^)	[11]
MI-mesoporous silica	Ratiometric fluorescence	0.01–5 μmol L^−1^	0.003 μmol L^−1^	Soil, plants	N (CdTe QDs)	[12]
Carbon dots (leafy veg.)	Fluorescence	0.037–0.2 mg L^−1^	0.00187 mg kg^−1^	Lettuce, spinach	Y	[13]
**DQBM-B**	Fluorescence (IFE)	0.375–4.50 μmol L^−1^	0.75 μmol L^−1^	Cucumber extract (spiked)	Y	This work

**Table 2:** Recovery of IMI in spiked cucumber extracts within the linear range of the probe (*n* = 3).

Spiked Concentration (μmol L^−1^)	Mean Detected Concentration (μmol L^−1^)	Average Recovery (%)	RSD (%)
0.375	0.380	101	8
2.25	2.29	102	2
4.50	4.75	106	5

## Data Availability

The data can be obtained from the author.

## Abbreviations

The following abbreviations are used in this manuscript:

**DQBM-B**	2,2′-((3-(1H-benzo[d]imidazol-2-yl)-1,2-phenylene)bis(oxy))bis(N-(quinolin-8-yl)acetamide)
ACQ	Aggregation-Caused Quenching
IMI	Imidacloprid
IFE	Inner Filter Effect
MRLs	Maximum Residue Limits
